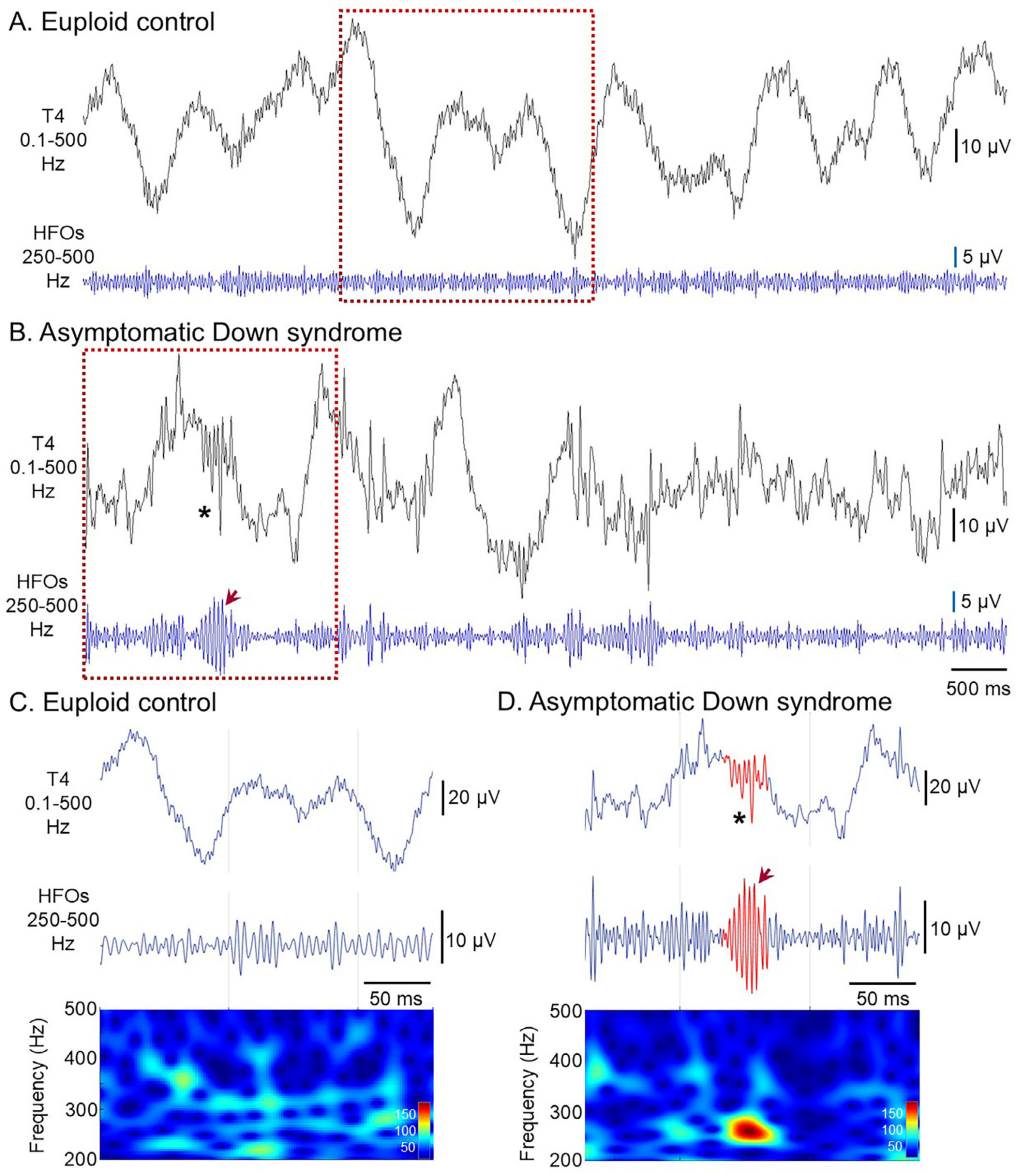# High Frequency Oscillations During Sleep in Down Syndrome

**DOI:** 10.1002/alz70856_105985

**Published:** 2026-01-07

**Authors:** Christos Panagiotis Lisgaras, Sandra Giménez, Maria Carmona‐Iragui, Lucía Maure‐Blesa, Esther M Blessing, Juan Fortea, Ricardo S. Osorio

**Affiliations:** ^1^ New York University Grossman School of Medicine, New York, NY, USA; ^2^ Nathan S. Kline Institute for Psychiatric Research, Center for Dementia Research, Orangeburg, NY, USA; ^3^ Multidisciplinary Sleep unit. Hospital de la Santa Creu i Sant Pau, Institut d'Investigació Biomèdica Sant Pau (IIB SANT PAU), Barcelona, Spain; ^4^ Sant Pau Memory Unit, Department of Neurology, Hospital de la Santa Creu i Sant Pau, Biomedical Research Institute Sant Pau, Universitat Autònoma de Barcelona, Barcelona, Spain; ^5^ Sant Pau Memory Unit, Hospital de la Santa Creu i Sant Pau, Biomedical Research Institute Sant Pau, Universitat Autònoma de Barcelona, Barcelona, Spain; ^6^ Barcelona Down Medical Center, Fundació Catalana Síndrome de Down, Barcelona, Spain; ^7^ Center for Biomedical Investigation Network for Neurodegenerative Diseases (CIBERNED), Madrid, Spain; ^8^ CIBERNED, Network Center for Biomedical Research in Neurodegenerative Diseases, National Institute of Health Carlos III, Madrid, Spain; ^9^ Sant Pau Memory Unit, Hospital de la Santa Creu i Sant Pau, Institut de Recerca Sant Pau ‐ Universitat Autònoma de Barcelona, Barcelona, Spain; ^10^ New York University, New York City, NY, USA; ^11^ Nathan S. Kline Institute, Orangeburg, NY, USA; ^12^ Sant Pau Memory Unit, Department of Neurology, Hospital de la Santa Creu i Sant Pau, Institut d'Investigació Biomèdica Sant Pau (IIB SANT PAU), Facultad de Medicina ‐ Universitat Autònoma de Barcelona, Barcelona, Spain; ^13^ Center of Biomedical Investigation Network for Neurodegenerative Diseases (CIBERNED), Madrid, Spain; ^14^ Center for Sleep and Brain Health, Department of Psychiatry, NYU Langone Health, New York, NY, USA; ^15^ NYU Grossman School of Medicine, New York, NY, USA

## Abstract

**Background:**

Alzheimer's disease (AD) dementia has near full penetrance in adults with Down syndrome (DS) and is strongly linked to late‐onset myoclonic epilepsy syndrome (LOMEDS). However, promising biomarkers of epileptogenicity, such as high frequency oscillations (HFOs>250Hz), have not been studied. This study is the first to use wideband polysomnography in DS to investigate if HFOs occurred and preceded AD dementia and LOMEDS.

**Method:**

Wideband (0.1‐500Hz, 2048Hz) polysomnography was performed using the international 10‐20 system. HFOs were automatically detected during slow‐wave sleep, followed by manual review.

**Result:**

Eleven individuals with DS and five age‐matched euploid controls were studied. HFOs were detected in all DS cases but not controls, with a mean rate of 7.4±2.0HFOs/min. HFOs emerged before AD dementia and LOMEDS diagnoses. A trend toward increased HFO rates with age in DS cases warrants further confirmation.

**Conclusion:**

HFOs are promising biomarkers that may predict symptomatic AD dementia in adults with DS.